# Supplementary figures and images for: Forage preference in two geographically co-occurring fungus gardening ants: A dietary DNA approach
Source: PLoS One. 2025 Dec 4;20(12):e0336150. doi: 10.1371/journal.pone.0336150 (PMC12677564; doi:10.1371/journal.pone.0336150)

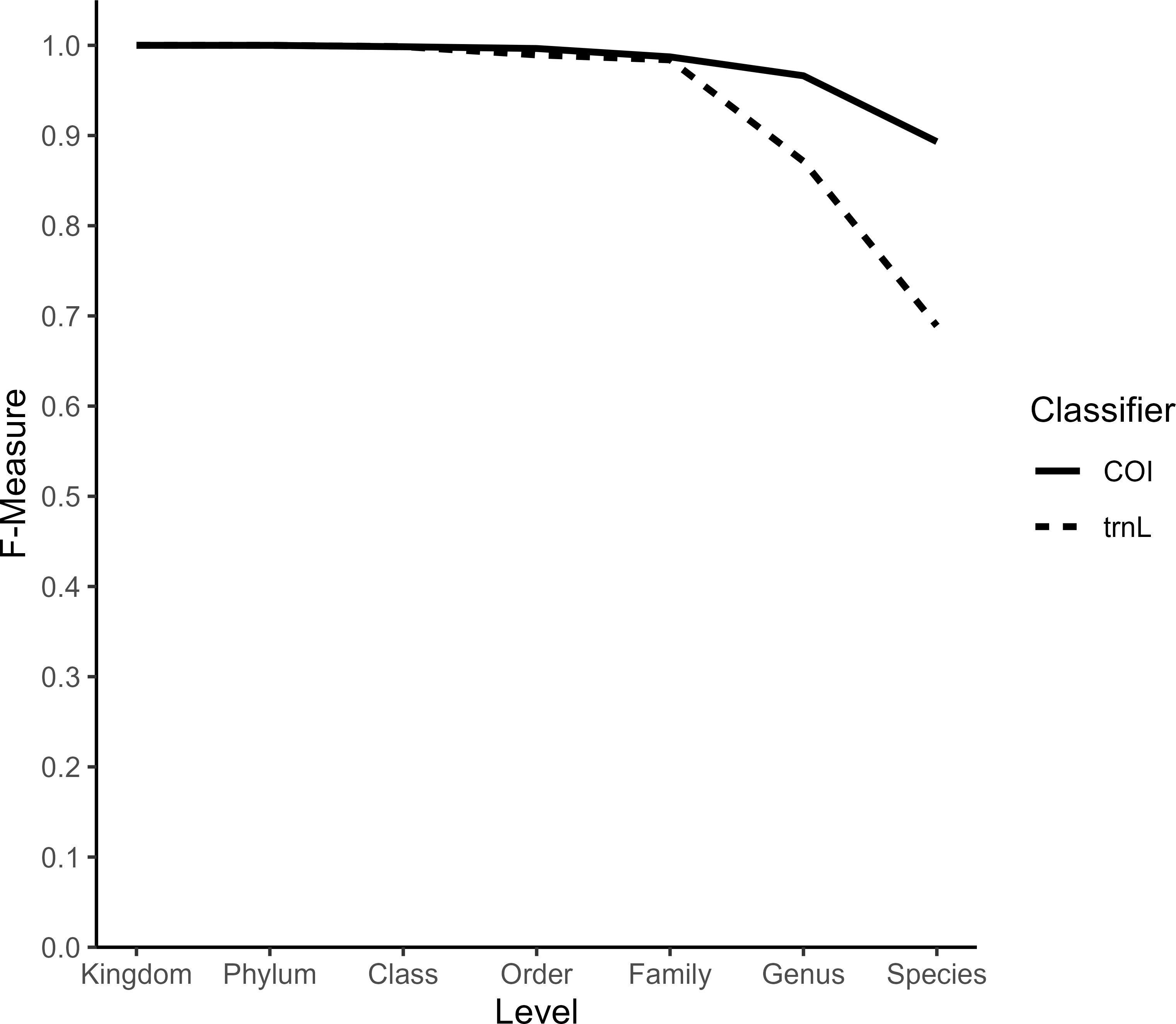

Supplement: S1 Fig — (TIF) [file pone.0336150.s001.tif]
